# Pinning down ploidy in paleopolyploid plants

**DOI:** 10.1186/s12864-018-4624-y

**Published:** 2018-05-08

**Authors:** Yue Zhang, Chunfang Zheng, David Sankoff

**Affiliations:** Department of Mathematics and Statistics, University of Ottawa, 585 King Edward, Ottawa, K1N 6N5 Canada

**Keywords:** Whole genome duplication, Gene loss, Birth and death process, Multinomial model, Paralog gene tree, Sequence divergence, *Brassica rapa*

## Abstract

**Background:**

Fractionation is the genome-wide process of losing one gene per duplicate pair following whole genome multiplication (doubling, tripling, …). This is important in the evolution of plants over tens of millions of years, because of their repeated cycles of genome multiplication and fractionation. One type of evidence in the study of these processes is the frequency distribution of similarities between the two genes, over all the duplicate pairs in the genome.

**Results:**

We study modeling and inference problems around the processes of fractionation and whole genome multiplication focusing first on the frequency distribution of similarities of duplicate pairs in the genome. Our birth-and-death model accounts for repeated duplication, triplication or other multiplication events, as well as fractionation rates among multiple progeny of a single gene specific to each event. It also has a biologically and combinatorially well-motivated way of handling the tendency for at least one sibling to survive fractionation. The method settles previously unexplored questions about the expected number of gene pairs tracing their ancestry back to each multiplication event. We exemplify the algebraic concepts inherent in our models and on *Brassica rapa*, whose evolutionary history is well-known. We demonstrate the quantitative analysis of high-similarity gene pairs and triples to confirm the known ploidies of events in the lineage of *B. rapa*.

**Conclusions:**

Our birth-and-death model accounts for the similarity distribution of paralogs in terms of multiple rounds of whole genome multiplication and fractionation. An analysis of high-similarity gene triples confirms the recent *Brassica* triplication.

## Background

While the genomic multiplicity of recent polyploids is accessible through cytogenetics and other methodologies, the nature of early large-scale genome events like auto- and allopolyploidization is obscured by interchromosomal translocations, chromosome fusions and other chromosomal rearrangements, by gene family expansions and fractionated gene loss and by sequence divergence between paralogs. One valuable line of evidence about these ancestral events is the discovery of two sets of at least four or five collinear pairs of highly related genes – paralogs – in close succession in different regions of the genome, known as paralogous synteny blocks. Insofar as all the paralog pairs in a paralogous synteny block resemble each other to the same extent, this indicates that there was a duplication of the chromosomal region containing them, which can then be dated approximately according to the degree of DNA sequence divergence. If there are many syntenic blocks of the same age throughout the genome, this is suggestive of a whole genome duplication at that point in time.

In 2007 Jaillon et al. noted that syntenic regions in the genome of grape (*Vitis vinifera*) were distributed as triples, not just duplicates [1]. Much of the genome could be partitioned into seven sets of three syntenic regions, indicative of a whole genome triplication over 100 M years ago, producing a 21-chromosome grape ancestor from a 7-chromosome precursor. Of interest is that in each triplet of regions, forming three pairs of regions, there were many duplicate gene pairs – involving just two regions – but very few actual triples of three highly related genes, one in each region. In addition there were very few duplications within a single region, or between genes in two different triplets among the seven sets of grape triplets of regions. The 21-chromosome construct has since been widely recognized as the ancestor of the core eudicots. The principle of three-way similarities among syntenic regions, indicated by

- some duplicated pairs between each two of the three regions,
- with or without any triplicated genes,
- no pairs within a single region and
- no pairs between different triples of regions,

is the signature pattern for ancient whole genome triplication, or paleohexaploidy. This may be generalized in straightforward ways to octoploidy and higher multiplicities of polyploidization. For example, an ancient octoploidization would be reflected in 4-tuples of regions, where the would be some duplicated gene pairs between each of the ${4\choose 2}=6$ pairs of regions, but no gene pairs within regions and no gene pairs between different 4-tuples.

Another type of important evidence in analyzing ancient polyploidization events is the distribution of coding sequence similarities between two paralogous genes. All flowering plants, and indeed most land plants, have at least one, and generally two, three or more polyploidizations in their history. The distribution of similarities is then a mixture of distributions, each of which is centered at a similarity value indicative of the age of one of the polyploidizations. We have developed a model for predicting the shape of these distributions based on the event times, the ploidy multiplicities of the events, rates of loss of duplicate genes from the genome (*fractionation*), and rates of sequence divergence [2]. This model produces a *paralog tree* in the form of a birth and death process with one biologically-motivated constraint, which remains mathematically tractable and whose parameters are well suited to statistical inference. Because of a trade-off between ploidy and fractionation rates, however, in many instances the multiplicity of the various ploidy events in the evolution of a genome cannot be determined uniquely, which is a severe problem for understanding its history.

One goal of this paper is to remedy this shortcoming by combining the syntenic approach pioneered in [1] with the paralog tree model of [2] to produce a method capable of estimating the multiplicity of the polyploidization events, as well as the fractionation parameters.

The next section summarizes the general model for generating the distribution of paralog similarities. This is followed by a brief section describing the inference of the parameters. We then focus on two particular instances, one where a hexaploidization (whole genome triplication) precedes a tetraploidization (whole genome duplication), and the other where the triplication follows the duplication. The difficulty of ploidy inference is illustrated with data from the turnip, or Napa cabbage (*Brassica rapa*) genome, and investigated in algebraic detail. In a section entitled “Counting triples”, we introduce the method inspired by [1] for distinguishing whole genome triplication from whole genome duplication, given the distribution of duplicate gene similarity, and we apply this to confirm the known sequence of events in the ancestral history of this species.

## Methods

### The general model

We summarize and correct a new and general model [3] for the repeated cycle of polyploidization events, each followed by fractionation. This model allows an arbitrary number of events and rates of fractionation of the progeny of any gene holding across the entire genome after each event. From this we calculate expected numbers of duplicate gene pairs, at the time of observation (i.e., the present time), originating at each of the historical polyploidization events, leading to the prediction of the entire distribution of similarities, using standard models of mutational processes.

The model is a continuous-time birth-and-death process with the entire population synchronized as to birth times and number of progeny, but with the number of deaths of the siblings in each individual “litter” determined probabilistically.

### The birth-and-death process

**Fig. 1:**
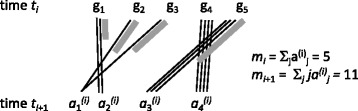
Event with ploidy *r*_*i*_=4, showing population of *m*_*i*_=5 genes at time *t*_*i*_, each giving rise to 4 progeny, of which 1≤*j*≤4 survive until time *t*_*i*+1_. $a_{j}^{(i)}$ is the number of times *j* progeny survive. In the diagram, thin solid lines represent individual progeny that survive, and thick grey lines represent the total progeny of a gene that do not survive. In this example the only gene, all of whose progeny survive, is *g*_4_. Here $a_{1}^{(i)}=2, a_{2}^{(i)}=a_{3}^{(i)}=a_{4}^{(i)}=1, m_{i+1}=2\times 1+1\times 2+1\times 3+1\times 4 = 11$

The process starts with *m*_1_≥1 genes at time *t*_1_; at times *t*_1_<⋯<*t*_*n*−1_ for some *n*≥1, each existing gene is replaced by *r*_1_,…,*r*_*n*−1_ progeny, respectively, where each *r*_*i*_≥2. As illustrated in Fig. 1, for each gene’s progeny, at least one and at most *r*_*i*_ genes survive until time *t*_*i*+1_, as governed by a probability distribution $u_{1}^{(i)},\dots,u_{r_{i}}^{(i)}$.

The results are observed at time *t*_*n*_, namely a measure of similarity (e.g., coding sequence similarity) between all pairs of genes in the population, where the *m*_1_ original genes are considered to be unrelated or too remotely related to be considered.

Let there be *m*_*i*_ genes at time *t*_*i*_, let $a_{1}^{(i)},\dots, a_{r_{i}}^{(i)}$ be the number of cases where 1,…,*r*_*i*_ copies survive fractionation until time *t*_*i*+1_, so that $\sum _{j=1}^{r_{i}}{a_{j}^{(i)}}=m_{i}$. Note that there is no provision for *g* to have zero surviving descendants (i.e., $a_{0}^{(i)} \equiv 0$); these genes would be considered as leaving no evidence for their existence and are not counted in *m*_*i*_. Note that that $m_{i+1} =\sum _{j=1}^{r_{i}}j{a_{j}^{(i)}}$.

We use $u_{j}^{(i)}$ to represent the probability that *j* of the *r*_*i*_ potential copies survive to time *t*_*i*+1_, for *j*=1,…,*r*_*i*_.

Thus the probability distribution of the evolutionary histories represented by **r**={*r*_*i*_}_*i*=1…*n*−1_ and the variable $\mathbf {a}=\left \{a_{j}^{(i)}\right \}_{j=1\dots r_{i}}^{i=1\dots n-1}$ is

1 $$ P\left(\mathbf{r;a}\right)= \prod_{i=1}^{n-1}\left[{m_{i}\choose{a_{1}^{(i)},\dots,a_{r_{i}}^{(i)}}}\prod_{j=1}^{r_{i}} \left(u_{j}^{(i)}\right)^{a_{j}^{(i)}}\right].  $$

The expected number of genes at time *t*_*n*_ is then

2 $$ \mathbf{E}(m_{n})=\sum_{\mathbf{a}}P(\mathbf{r;a})\ m_{n}.  $$

Similarly, we write

3 $$ P^{(j,k)}\left(\mathbf{r;a}\right)= \prod_{i=j}^{k-1}\left[{{m_{i}}\choose{a_{1}^{(i)},\dots,a_{r_{i}}^{(i)}}}\prod_{h=1}^{r_{i}} \left(u_{h}^{(i)}\right)^{a_{h}^{(i)}}\right]  $$

for the probability measure over all events starting at time *t*_*j*_ with *m*_*j*_ genes, and preceding time *t*_*k*_. In this case the expected number of genes at time *t*_*k*_ is

4 $$ \mathbf{E}^{(j,k)}(m_{k})=\sum_{\mathbf{a}}P^{(j,k)}(\mathbf{r;a})\ m_{k}.  $$

### The paralog pairs

**Fig. 2:**
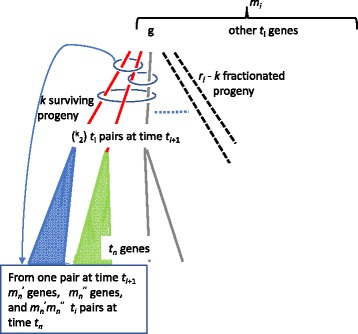
Counting *t*_*i*_-pairs. The three unfractionated progeny of gene *g* define three *t*_*i*_-pairs, as indicated by three ovals. We follow the pair contained in the uppermost oval, as the two members at time *t*_*i*+1_ independently (shaded triangles) evolve into $m^{\prime }_{n}$ and *mn*″ genes, respectively, at time *t*_*n*_, defining $m^{\prime }_{n}m''_{n} t_{i}$-pairs at time *t*_*n*_

Having characterized the origin and survival of individual genes and their descendants in the environment of recurrent polyploidization and fractionation, we can now focus on the pairs of genes observed at time *t*_*n*_. Our discussion is illustrated by Fig. 2.

For each of the $a_{j}^{(i)}$ genes with *j* surviving copies, *j*≥2, there are ${{j}\choose {2}}$ surviving pairs of genes. If *j*=1 there are no pairs. The total number of pairs created at time *t*_*i*_ and surviving to time *t*_*i*+1_ is thus

5 $$ d^{(i,i+1)}=\sum_{j=2}^{r_{i}}{{j}\choose{2}}a_{j}^{(i)}.  $$

These are called the *t*_*i*_-pairs at time *t*_*i*+1_. The expected number of such pairs is

6 $$ {\mathbf{E}}\left(d^{(i,i+1)}\right)=\sum_{\mathbf{a}}P^{(1,i+1)}(\mathbf{r;a})\sum_{j=2}^{r_{i}}{{j}\choose{2}}a_{j}^{(i)}.  $$

At time *t*_*j*_,for i + 1≤*j*≤*n*, any two descendants of the two genes making up a *t*_*i*_-pair *with no more recent common ancestor* is also called a *t*_*i*_-pair (at time *t*_*j*_). In other words, for any two genes at time *t*_*j*_, they form a *t*_*i*_-pair if their most recent common ancestor underwent polyploidization at time *t*_*i*_.

For a given *t*_*i*_-pair *g*^′^ and *g*^″^ at time *t*_*i*+1_, where *i*<*n*−1, the expected number of pairs of descendants *d*^(*i*,*n*)^ having no more recent common ancestor than *g*^′^ and *g*^″^, will be

7 $$ {\mathbf{E}}\left(d^{(i,n)}\right)={\mathbf{E}}\left(d^{(i,i+1)}\right)\left(\mathbf{E}^{(i+1,n)}(m_{n})\right)^{2}  $$

where *m*_*i*+1_=1. This follows from the independence of the fractionation process between time *t*_*i*_ and time *t*_*i*+1_ and both parts of the process starting with *g*^′^ and *g*^″^.

Not all the *m*_*n*_ genes in Eq. (2) are in pairs. Because of fractionation after every polyploidization event, some genes will remain unpaired. We have

8 $$\begin{array}{*{20}l} m^{*}&\in\{0,\ldots,m_{1}\} \\ {\mathbf{E}}(m^{*})&=m_{1} \prod_{i=1}^{n-1} u^{(i)}_{1}, \end{array} $$

where *m*^∗^ is the current number of unpaired genes.

The terms **E**(*d*^(*i*,*i*+1)^) and **E**^(*i*+1,*n*)^(*m*_*n*_) in Eq. (7) both involve calculating probabilites with Eq. (3). As *n* and the *r*_*i*_ increase, so do the *m*_*i*_, and this becomes computationally very expensive, due to the product of multinomial coefficients in Eq. (3) and the sum of many such probabilities in Eq. (4). Nevertheless, making use of the recursive nature of these calculations allows for more efficiency than the explicit generation of evolutionary histories and the counting of pairs within each one.

### The Distribution of Similarities

Knowing the expected number of pairs of genes originating at each WGD in the past is the first step in predicting the full distribution *F* of similarities. The second step is to derive the actual distribution of gene pair similarities, or an appropriate approximation to it, for *t*_*i*_-pairs.

One way gene pair divergence may be measured is in terms of a probability *p* reflecting *similarity* – the proportion of nucleotide positions that are occupied by the same base in the two orthologs (or paralogs).

Besides *p*, the other important parameter is *G*, reflecting average gene length in terms of the number of nucleotides in the genes’ coding region. Because this length varies greatly, in practice *G* needs to be estimated.

In the simplest case, the distribution of similarities is the binomial B(*G*,*p*_*i*_), where

9 $$ p_{i}=\frac{1}{4}+\frac{3}{4}\mathrm{e}^{-\lambda t_{i}}\in [\!0,1],  $$

and is related to the time *t*_*i*_∈[ 0,*∞*) elapsed since the event that gave rise to the pair. This distribution has

10 $$\begin{array}{*{20}l} \text{mean}:\ \ \mu_{i}&= {Gp}_{i} \\ &=G\left(\frac{1}{4}+\frac{3}{4}\mathrm{e}^{-\lambda t_{i}}\right)\in [0,1]\\ \text{variance}: \ \ \sigma_{i}^{2}&={Gp}_{i}\left(1-p_{i}\right)  \\ &=\frac{3}{16}G\left(1+3\mathrm{e}^{-\lambda t_{i}}\right)\left(1-\mathrm{e}^{-\lambda t_{i}}\right),  \end{array} $$

where *λ*>0 is a divergence rate parameter.

The densities of similarities of *t*_*i*_-pairs can be approximated by a normal distribution ${\mathbf {N}}\left (\mu _{i},\sigma _{i}^{2}\right)$ (as long as *p*_*i*_ is not too close to 1.0), and the expected frequency by

11 $$ F_{i} = {\mathbf E}\left(d^{(i,n)}\right){\mathbf{N}}\left(\mu_{i},\sigma_{i}^{2}\right).  $$

We can predict the entire frequency distribution over all *t*_*i*_ as:

12 $$ F(t)=\sum_{i=1}^{n-1}F_{i}(t),  $$

keeping in mind that the model also predicts unpaired genes according to Eq. (8). Then the predicted relative frequencies become

13 $$\begin{array}{*{20}l} q(i)&=\frac{F(i)}{\mathbf{E}(m^{*})+\sum_{j}F(j)}  \\ q^{*}&=\frac{\mathbf{E}(m^{*})}{\mathbf{E}(m^{*})+\sum_{j}F(j)}. \end{array} $$

### Inference

The distribution of gene pair similarities is of the form *f*(*k*), where *k*=*k*_min_,…,*k*_max_. The data may also include *f*^∗^, the frequency of unpaired genes. The value of *k*_min_ is set to eliminate pairs due to noise or to polyploidization events earlier than those of immediate interest. At the other extreme, *k*_max_ is set somewhat lower than 100%, in order to remove any effects of heterozygosity, whereby an apparent duplicate gene pair actually consists of two alleles of a single gene, rather than two genes at different positions in the genome.

The likelihood of a model, given some data set is

14 $$ L=C\prod_{i=k_{\text{min}}}^{k_{\text{max}}}q(i)^{f(i)}(q^{*})^{f^{*}},  $$

where *q* depends only on the parameters of the model, and the maximum likelihood estimators of the parameters of a model can be found by maximizing

15 $$ f^{*}\log q^{*}+\sum_{i=k_{\text{min}}}^{k_{\text{max}}}f(i) \log q(i)  $$

with respect to these parameters.

## Results and discussion

### Two models for one dataset

For a given instance of the above model, if we know some of the parameters, we can infer the others. This includes

- the *t*_*i*_: the times of each event,
- the fractionation rates,
- *λ*: the divergence rate, and
- *G*: the gene length parameter.

**Fig. 3:**
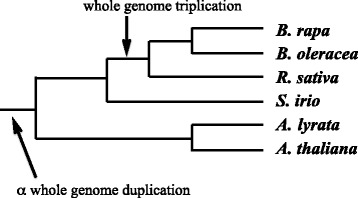
Evolutionary history of the Brassicales, showing the *α* genome duplication in the lineage of all the species and the more recent *Brassica* triplication

However, we cannot easily estimate the *r*_*i*_, the event ploidies, from the distribution of paralog pair similarities. To understand why, we consider an important example, namely the outcome of two events, a tetraploidy leading to a whole genome duplication and hexaploidy, leading to a whole genome triplication. We will illustrate with data on the *Brassica rapa* genome [4], member of the *Brassica* genus, known to have undergone a triplication after an earlier duplication shared with other Brassicales genera, such as *Arabidopsis*, as shown in Fig. 3.

**Fig. 4:**
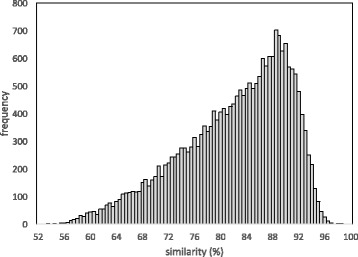
Duplicate gene similarities in syntenic blocks in the *Brassica rapa* genome self-comparison

The distribution of gene pair similarities derived from SYNMAP (on the COGE platform [5, 6]) is shown in Fig. 4. Only the recent event, the *Brassica* triplication, is clearly visible as a distinct peak in the histogram, but the voluminous tail at early similarities attests to the effect of the earlier Brassicales duplication. Brassicales is a rosid order and as such also descends from the *γ* core eudicot triplication, which would have produced pairs with around 70% similarity, but very few remained in synteny blocks, so for the purposes of our subsequent analysis, we ignore this event. Indeed, we imposed no bounds *k*_min_ or *k*_max_ on the data produced by SYNMAP.

We can explore the discriminatory power of our method by fitting two models to these data, one where a whole genome duplication precedes a triplication, known to be true, and an incorrect one where the duplication follows the triplication.

**Fig. 5:**
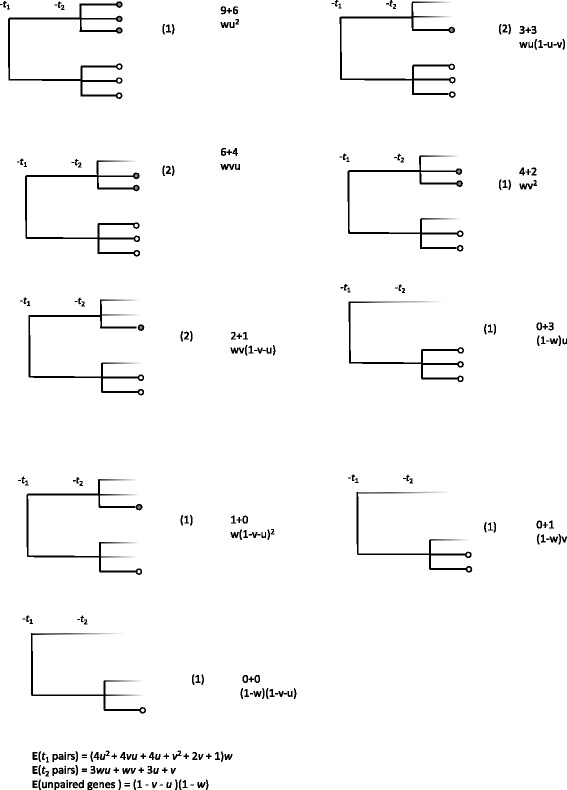
All paralog trees generated by a genome duplication at time *t*_1_ followed by a triplication at time *t*_2_, both events followed by fractionation. Present-day genes shaded according to most recent event. Numbers above probability are counts of *t*_1_+*t*_2_ pairs, and numbers in parentheses count the number of different trees (only one shown) with the same structure

**Fig. 6:**
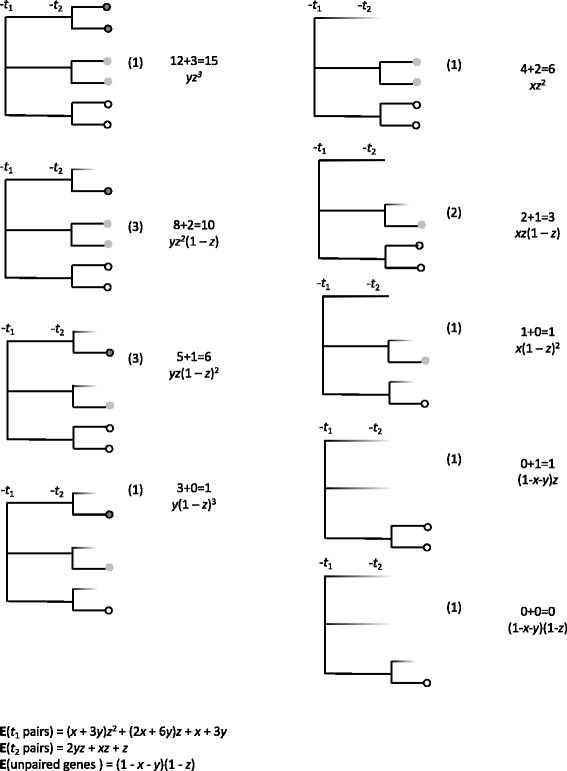
All paralog trees generated by a genome triplication at time *t*_1_ followed by a duplication at time *t*_2_, both events followed by fractionation. Present-day genes shaded according to most recent event. Numbers above probability are counts of *t*_1_+*t*_2_ pairs, and numbers in parentheses count the number of different trees (only one shown) with the same structure

The calculations leading to Eqs. (7) and (8) are not lengthy in the case of these two models, and are portrayed schematically in Figs. 5 and 6, where *u*,*v*,*w*,*x*,*y* and *z* are probabilities that can be fixed independently of each other, as long as *u*+*v*<1 and *x*+*y*<1. (To avoid trivial models in either case, *u*,*w*,*x* and *z* must be greater than zero and less than 1, while *v* and *y* must be greater or equal to zero and less than 1. We will term these *valid models*.)

In Fig. 5, $w=u_{2}^{(1)}$, the probability that both offspring survive until time *t*_2_ after the first duplication at time *t*_1_, so that 1−*w* is the probability that only one survives. After the triplication at time *t*_2_, the probabilities are $u=u_{3}^{(2)}$ and $v=u_{2}^{(2)}$ that three offspring or two offspring, respectively, survive until time *t*_3_.

Looking at the second paralog tree in the left-hand column of Fig. 5, for example, Eq. (1) becomes

16 $$\begin{array}{*{20}l} P(\mathbf{r;a})&= \prod_{i=1}^{n-1}\left[{{m_{i}}\choose{a_{1}^{(i)},\dots,a_{r_{i}}^{(i)}}}\prod_{j=1}^{r_{i}} \left(u_{j}^{(i)}\right)^{a_{j}^{(i)}}\right]  \\ &={{1}\choose{a_{1}^{(1)},a_{2}^{(1)}}}w^{a_{2}^{(1)}}(1-w)^{a_{1}^{(1)}} {{2a_{2}^{(1)}+a_{1}^{(1)}}\choose{a_{1}^{(2)},a_{2}^{(2)},a_{3}^{(2)}}}\\ &\quad\times u^{a_{3}^{(2)}}v^{a_{2}^{(2)}}(1-u-v)^{a_{1}^{(2)}} \\ &={{1}\choose{0,1}}w{{2}\choose{0,1,1}}uv \\ &=2wvu. \end{array} $$

The coefficient 2 in this expression corresponds to the two “versions" of the diagram with the colours of the gene pair and gene triple permuted. Note that the branches without coloured dots in most of the paralog trees are simply meant to be suggestive of the fractionation process, do not reflect anything in Eq. (1), and are not involved in the colour permutations in counting the number of versions. The number of *t*_1_ and *t*_2_ pairs at time *t*_3_, as calculated in Eqs. (5-8), can be counted directly for each tree in the figure.

Turning to Fig. 6, $z=u_{2}^{(2)}$, the probability that both offspring survive until time *t*_3_ after the second event (duplication) at time *t*_2_, so that 1−*z* is the probability that only one survives. After the triplication at time *t*_1_, the probabilities are $x=u_{3}^{(1)}$ and $y=u_{2}^{(1)}$ that three offspring or two offspring, respectively, survive until time *t*_2_.

The expected number of pairs in the duplication precedes triplication model (Fig. 5) is given by:

17 $$\begin{array}{*{20}l} {\mathbf{E}}\left(t_{1}\ \text{pairs}\right) &=\left(4u^{2}+4uv+4u+v^{2}+2v+1\right)w \\ {\mathbf{E}}(t_{2}\, \text{ pairs}) &= 3wu+wv+3u+v \\ {\mathbf{E}}(\text{unpaired}) &= (1-w)(1-u-v).  \end{array} $$

The same quantities in the triplication-first model (Fig. 6) are:

18 $$\begin{array}{*{20}l} {\mathbf{E}}(t_{1}\ \text{pairs})&= (x+3y)(1+z)^{2}\\ {\mathbf{E}}(t_{2}\ \text{ pairs})&= 2yz+ xz+z  \\ {\mathbf{E}}(\text{unpaired}) &= (1-z)(1-x-y)  \end{array} $$

Can the principle of maximum likelihood discriminate between the two models? The likelihood of either model depends only on the *q*(*i*) and *q*^∗^ in Eq. (14). Then the parameters of one model can be related to a set of parameter values *in the complex field* (i.e., not necessarily probabilities) with the same likelihood in the other model through the equations:

19 $$\begin{array}{*{20}l} \left(4u^{2}+4uv+4u+v^{2}+2v+1\right)w&=(x+3y)(1+z)^{2}\\ 3wu+wv+3u+v&=2yz+ xz+z\\ (1-w)(1-u-v)&=(1-z)(1-x-y), \end{array} $$

This implies that all maximum likelihood solutions in both models have the same likelihood. Furthermore, for large enough samples we can expect at least one maximum likelihood solution in each valid model. Because the parameters are underdetermined, the likelihood depending only on the *q*(*i*) and *q*^∗^ and not the absolute frequencies *f*(*i*) and *f*^∗^, there may be several solutions. In addition, if in one model the maximum likelihood solution involves parameters which satisfy the conditions of a valid model, this is not necessarily true of the corresponding parameters in the other model.

We may then ask, do Eqs. (17) and (18) determine a bijection between some valid model in (*u*,*v*,*w*) space and some valid model in (*x*,*y*,*z*) space? The answer is determined by the intersection of (*u*,*v*,*w*)∈[ 0,1]^3^∩{*u*+*v*<1} and (*x*,*y*,*z*)∈[ 0,1]^3^∩{*x*+*y*<1} and the algebraic variety determined by the system in Eq. (19).

**Fig. 7:**
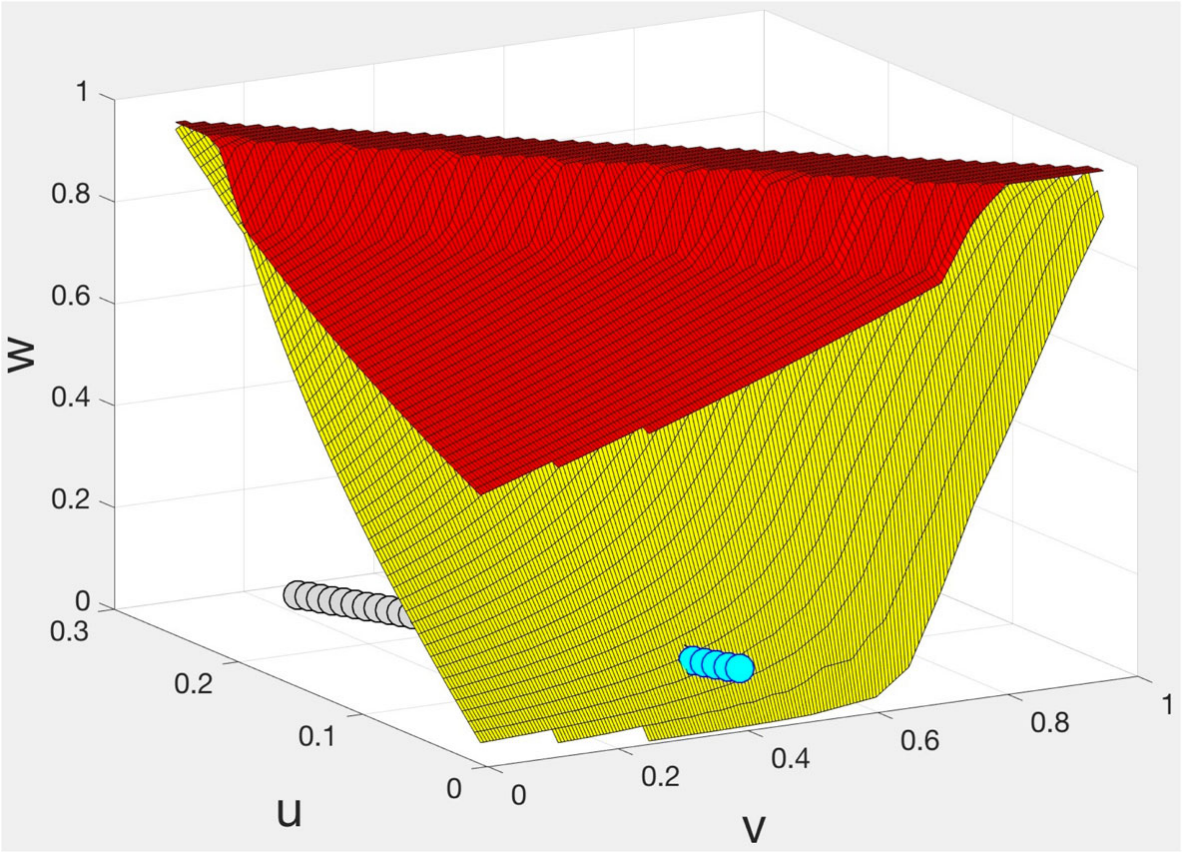
(*u*,*v*,*w*) space with MLE solutions for (*x*,*y*,*z*) model (blue dots) and (*u*,*v*,*w*) model (blue and grey dots). Corresponding (*x*,*y*,*z*) models exist only for the (*u*,*v*,*w*) values in the volume bounded by the red and yellow sheets

By systematically exploring a three-dimensional grid in each cube, we located all points in the valid region of the (*u*,*v*,*w*) cube for which Eq. (19) produced points in the valid region of the (*x*,*y*,*z*) cube. This produced two surfaces as depicted in Fig. 7 between which the two models have a correspondence. Outside of this volume, Eq. (19) have only solutions that are complex or outside one or both valid regions.

In Fig. 7, we see that the multiple maximum likelihood solutions for the *B. rapa* data in (*u*,*v*,*w*) space form a linear subspace, only part of which also contains solutions for the (*x*,*y*,*z*) model.

To restrict the set of solutions, we may make use of *f*^∗^, the observed number of unpaired genes, which is not directly involved in the likelihood maximization - only *q*∗ is. This number is 17,751. Unfortunately, we have no access to the number of genes in the ancestral genome preceding the two polyploidization events, but we can guess, based on core eudicots that have escaped polyploidization after *γ*, such as grape [1] and Robusta coffee [7], that 25,000 is a reasonable value. Of genomes that have polyploidized and fractionated, many, such as *Utricularia*, papaya, *Mimulus* or most pertinent, *Arabidopsis*, have returned currently to gene numbers less than 29,000 [8]. This means that of 25,000-29,000 ancestral genes, the average number of currently unpaired genes per original gene is 0.61-0.71. In Fig. 7, the grey dots at the extreme left represent valid solutions in the (*u*,*w*,*v*) space but not the (*x*,*y*,*z*) space, predicting 0.65-0.68 unpaired genes while for the blue dots corresponding to valid solutions in both spaces the predicted number is only 0.53-0.55, suggesting an ancestral gene complement of 32,000-34,000, which seems unlikely. These calculations thus lend more credence to the model where duplication precedes triplication.

In this model, we have used *u* and *v* as two independent parameters controlling fractionation for the triplication event, and similarly *x* and *y* in the triplication-first model. This may represent excessive parametrization, however, since there are very likely biological constraints on such pairs of parameters, though this has not yet been modeled or studied empirically. A reasonable way of modelling this is to postulate a constrained binomial process for the fractionation loss of one or two genes of each triple generated by the triplication event. Thus we may replace *u* and *v* by using a single parameter *s* and replace *x* and *y* by a single parameter *h* as follows:

20 $$\begin{array}{*{20}l} u=\frac{s^{2}}{3(1-s)+s^{2}}, &\qquad v=\frac{3s(1-s)}{3(1-s)+s^{2}} \\ x=\frac{3h(1-h)}{3(1-h)+h^{2}}, &\qquad y=\frac{h^{2}}{3(1-h)+h^{2}}.  \end{array} $$

The investigation into the connection between the two models starts with

21 $$\begin{array}{*{20}l} \frac{9w}{\left(3-3s+s^{2} \right)^{2}}& = \frac{\left(3h+6hz+3hz^{2}\right)}{\left(3-3h+h^{2} \right)}\\ \frac{(3ws+3s)}{\left(3-3s+s^{2} \right)}&= \frac{3z}{\left(3-3h+h^{2} \right)} \\ \frac{(1-w)\left(3(1-s)^{2}\right)}{\left(3-3s+s^{2} \right)}&= \frac{(1-z)\left(3(1-h)^{2}\right)}{\left(3-3h+h^{2} \right)}  \end{array} $$

There being only two parameters in each model, we can use only two of the Eqs. (21) to understand the correspondences between these models. In this case there is a bijection between (*s*,*w*)∈[ 0,1]^2^ and (*h*,*z*)∈[ 0,1]^2^. However, corresponding points in the two spaces reflect different numbers of unpaired genes (and of *t*_1_ pairs and *t*_2_ pairs).

**Fig. 8:**
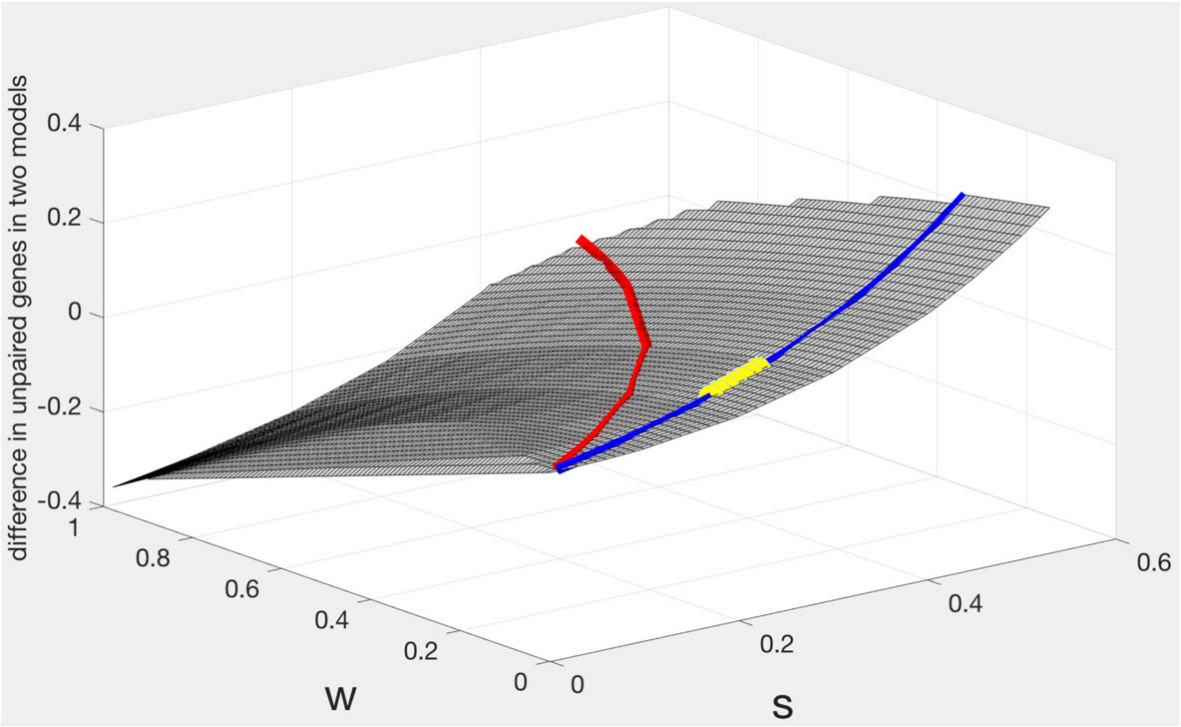
(*s*,*w*) surface showing the difference in the number of unpaired genes between corresponding points in the two models. The red line indicates points where the two models agree on the number of genes, but this does not intersect with the set of MLE solutions on the blue line). In particular, the yellow dots indicating realistic numbers of unpaired genes are far from the red line

The vertical axis in Fig. 8 represents the difference between the predictions of unpaired genes in the two models, with the red line tracing the values where the difference is zero. The blue line represents the maximum likelihood solutions for the *B. Rapa* data. The yellow dots identify (*s*,*w*) solutions that predict 0.6-0.68 currently unpaired genes per ancestral gene. Far from the red line, we conclude that the triplication-first model does not represent reality.

### Counting triples

We have seen that in contrast to the divergence and fractionation parameters, the ploidy of whole genome multiplication events is not easy to infer from the distribution of gene pair similarities. There are more direct ways, however, to establish the ploidy of these events. Most obvious in our case is the detection of highly similar, i.e., recently diverged, triples of genes or triples of chromosomal regions, as evidence of the late triplication model.

**Fig. 9:**
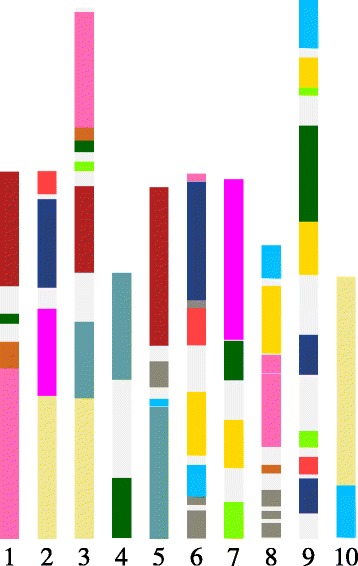
Regions of the 10 *Brassica rapa* chromosomes coloured by 14 triples of recently diverged regions. Grey regions: triple undetermined

The total number of genes in the CoGe *B. rapa* data set is 41,020. The application of SYNMAP to compare *B. rapa* with itself (cf. Fig. 4) shows there are 14 triples of paralogous regions made of long synteny blocks with relatively little interruption. These regions cover 80% of the genome, as can be seen in Fig. 9. For some of these triples, one or more of the three regions are divided among two or three chromosomes, due to genome rearrangement processes. But they all display the signature pattern of recent triplication enunciated by Jaillon et al. [1], namely large numbers of highly similar gene pairs *among* the three regions and relatively few highly similar gene pairs *within* each region, or between the regions and other parts of the genome.

The total number of gene pairs detected by SYNMAP is 22,406, including 13,716 (61%) where the members are in two paralogous regions and have high similarity, defined as 81% or higher. Of these, for the overwhelming majority, both members are in the 14 triples of regions.

Looking at all the high-similarity pairs, a large number of these form gene triples, 2392 of them, that are not part of a 4-tuple or higher. The great majority of these gene triples, 2118, are located in the 14 triples of high-similarity regions.

We can contrast this situation with the predictions of the triplication-first model. If the time of the duplication corresponded to *p*=0.87, then there would be many pairs with similarity >0.81, but few triples where all the pairs satisfied >0.81. There would, however, be many triples where all three pairs had similarity <0.81. This is clearly not the case.

## Conclusion

The decomposition of gene pair similarity distributions into a number of normal distributions has been staple of comparative genomics. Statistical mixture of distributions methods [9] have been used extensively, to detect the distributions, to find their means and to test their significance. Because these are general methods, they do not take into account the biological processes that gave rise to the distribution and thus may lead to meaningless results. For example, they can find significance in a two-distribution decomposition, in which the one reflecting an earlier event has smaller variance than the most recent one, a biological impossibility. They can produce a decomposition where a peak with a large amplitude is succeeded by one with very small amplitude, again biologically implausible.

For distributions reflecting genuine events, mixture methods may provide accurate measures of the timing of these events, but offer little else of biological interest. Our models go further, allowing, for the first time, the estimation of fractionation rates from pair similarity distributions. We have proposed algebraic machinery for comparing competing models, and as an illustrative test, used it to confirm what was already well-known, that the *B. rapa* genome triplication is more recent than its duplication event.

At the end we must conclude, despite the unexpected insights provided by mathematically modeling the genome multiplication-fractionation cycle, that to decide on the ploidy of the multiplication events, the strongest evidence, at least for the most recent events, is found in the tabulation of high-similarity pairs, triples, or other multiples. If few of the high-similarity pairs are in triples or other tuples, then the most recent event is likely to have been a tetraploidization. If a large proportion of the pairs are in triples but not in higher tuples, the event must have been a hexaploidization.

By judiciously parsing the similarity axis using cut-off values between peaks of the distribution, or between the mean values of inferred normal components of the overall distribution, we might hope in some cases to extend this simple approach to find the multiplicity of the earlier events,
